# Hidradenitis Suppurativa in Elderly Patients: Clinical and Therapeutical Outcomes—A Review of the Literature

**DOI:** 10.3390/medicina60091465

**Published:** 2024-09-06

**Authors:** Fabrizio Martora, Nello Tommasino, Claudio Brescia, Luca Potestio, Teresa Battista, Matteo Megna

**Affiliations:** Section of Dermatology, Department of Clinical Medicine and Surgery, University of Naples Federico II, Via Pansini 5, 80131 Napoli, Italy; nello.tommasino@libero.it (N.T.); claudio.brescia.1997@gmail.com (C.B.); potestioluca@gmail.com (L.P.); teresabattista12@gmail.com (T.B.); mat24@libero.it (M.M.)

**Keywords:** hidradenitis suppurativa, treatment, elderly, outcome

## Abstract

The management of hidradenitis suppurativa (HS) in elderly patients presents unique challenges due to its chronic inflammatory nature, heterogeneous clinical presentation and comorbidities. While HS typically affects the anogenital and intertriginous regions, elderly patients may exhibit atypical features such as the involvement of the neck, mammary area and gluteal region. The prevalence of HS in the elderly population is lower and the average age of disease onset is higher than in patients under 65. In contrast, it is unclear whether HS in the elderly has different clinical features. The elderly frequently present multiple comorbidities, including obesity, diabetes, and heart disease, which further complicate management decisions. Therapeutic interventions must consider the frailty and increased risk of multimorbidity and adverse events in elderly patients. While systemic antibiotics remain a mainstay of HS treatment, biologic agents such as TNFα inhibitors and secukinumab offer promising options for refractory cases. However, their safety and efficacy in elderly patients, particularly those with multiple comorbidities, require careful consideration. A comprehensive approach to managing HS in elderly patients involves not only pharmacological interventions but also lifestyle modifications and surgical options where appropriate. Multidisciplinary collaboration between dermatologists, geriatricians and other specialists is essential for tailoring treatment strategies and optimizing long-term outcomes and quality of life in special population.

## 1. Introduction

Hidradenitis suppurativa (HS) is a chronic, non-infective inflammatory disease that usually involves the anogenital and intertriginous regions of the body. The major clinical feature is the presence of papules and/or deep-seated painful nodules that show a slow tendency to spontaneous healing and progress to abscess-like lesions that drain foul-smelling purulent material. Furthermore, since the disease is typically relapse-remitting, new lesions may develop in adjacent sites, leading to developing sinus tracts, fistulas and hypertrophic scars that deserve a surgical resolution.

HS typically occurs after puberty, with the average age of onset in the second and third decades of life [1]. HS onset in elderly is very uncommon, and studies on this fragile class of patients are lacking.

Van Den Weijden et al. suggest defining HS in the elderly as HS tarda and to subdivide it as late onset if it develops after 60 years of age and persistent HS tarda if it has an earlier onset and persists after 60 years [2]. It is interesting to note that the HS female to male ratio is 3.3:1.0 [3], while in the older population a lower female to male ratio is observed (1.7:1.0); this might be due to the role of menopause on HS development and course [2]. Additionally, the clinical presentation may be different in elderly patients. For example, in the cohort of patients studied by Cazzaniga et al., the neck and mammary regions were more likely involved in older people [4], whereas the armpits and buttocks were less commonly involved compared to younger patients [2]. Since HS is a multifactorial disease, both genetics and lifestyle factors, in particular body mass index (BMI) and cigarette smoking, play a key role for HS development and flare-ups [5]. Family history for HS is typically associated with an earlier onset of clinical presentation [4]; on the other hand, older patients with HS are more likely to be current or ex-smokers [6]. Furthermore, there is evidence that current smokers, especially older patients, have a more severe form of the disease and may be less likely to respond to medical therapies [7]. Blum et al. found that Hurley 3 stage disease is more prevalent in older adults, with more sinus tracts and larger abscesses [6]. Population aging is notably increasing worldwide: more than 2 billion people are estimated to be older than 65 years old by 2050 [8], so third millennium medicine will be focused on the management of patients with several comorbidities and polypharmacotherapy. In this regard, old patients with HS have an increased risk of developing chronic obstructive pulmonary disease, intestinal bowel diseases, cardiopathy, diabetes and kidney dysfunction [2]. Hence, the aim of this review is to focus on HS in the elderly at both clinical and therapeutic levels.

## 2. Materials and Methods

This study is a narrative review; we conducted a narrative analysis and covered a broad range of topics by using studies of various complexity and design.

Pubmed, Cochrane Skin and Embase databases were reviewed up to March 2024. Search terms included “hidradenitis suppurativa”, “diagnosis”, “elderly”, “smoking”, “old age”, “biological therapies”, “geriatric”, “translational studies”, in the abstract and title. Manuscripts were found, screened, analyzed for relevant data and compared further. Only English language manuscripts were included in this study. This article is based on previous studies and does not include any studies of human participants or animals performed by any of the authors. The criteria for inclusion were English-language articles, empirical studies, and review papers to provide context for our findings. We reviewed the articles and omitted those with restrictive medical content.

**Figure 1:**
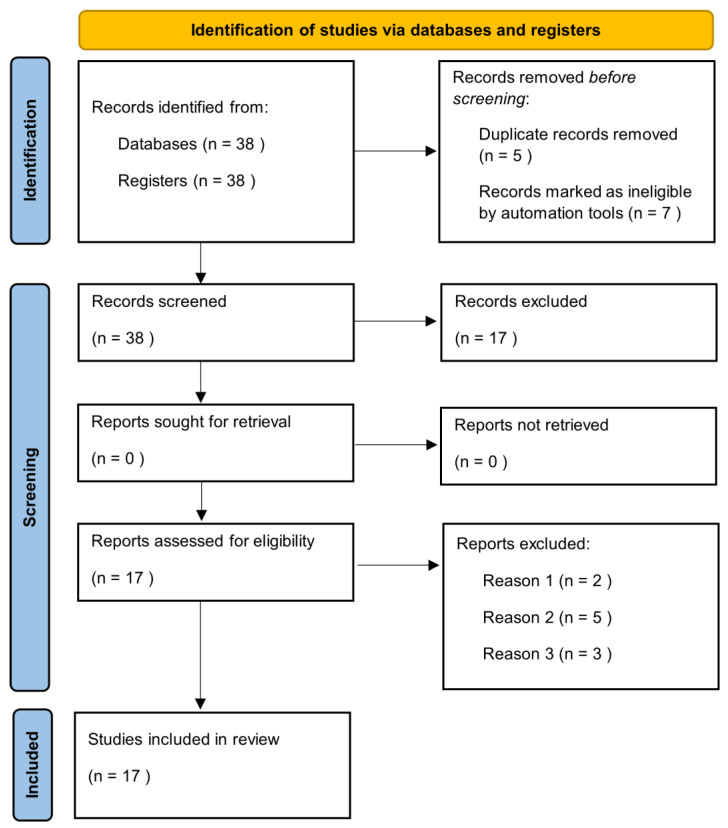
Prisma check list.

Manuscripts were identified, screened and extracted for relevant data following the PRISMA (Preferred Reporting Items for Systematic Reviews and meta-analyses) guideline (Figure 1).

## 3. Results

The literature search highlighted 38 articles, of which 17 satisfied the selection criteria for this narrative review. The results section has been divided into sub-sections in which the epidemiological and clinical characteristics of the elderly with HS and the impact of treatment on this category of patients are described.

### 3.1. Epidemiology

HS is an under-diagnosed disease, which affects approximately 1% of the world population but the exact prevalence in the general population is reported to be very variable. A retrospective study of 48 million people in the U.S. calculated a prevalence of 0.1%, but other works proposed values as high as 4.1% [9]. HS is four times more common in women and affects black and biracial people more frequently [9,10]. The mean age of onset is between the second and third decade, but it can appear at any age [11]. Two peaks of onset are estimated, in the late teens and around 40 years of age [12]. In total, 7.7% of patients with HS are under 13 years of age and knowledge on pediatric pathology is steadily increasing [13]. In contrast, data on HS in elderly population are scant. Among these people, the prevalence of HS is estimated at 0.8% [14]. According to Blum et al., the average age of disease onset is 41.8 in patients over 65 years, which is significantly higher than patients under 65 years, where the average is 20.1 [6]. Furthermore, in elderly HS, a reduction in the female–male ratio to 1.6 is observed [7] probably due to the menopause, which is associated with a decrease in the frequency of the condition [15]. For this reason, van der Weijden et al. defined the HS of the elderly, or tarda, from the age of 60, considering the menopause to be between 40 and 58 years. According to their estimates, the average age onset of HS was significantly higher in the elderly group than in the adult group (40 vs. 23), confirming the data of Blum et al. [2].

### 3.2. Clinical Features

Van der Weijden et al. also proposed a classification of HS tarda into late-onset HS, if it occurred after the age of 60, and persistent HS, if it began before the age of 60 and lasted beyond this age [2]. They found no clinical differences between these two categories, while there were some significant ones between adult and elderly patients. Indeed, among the elderly, the most affected sites were not those typical of adult HS, such as armpits, groin and buttocks (OR 0.595, 0.643 and 0.563 respectively), but of others (OR 1.887) [2]. They also investigated familiarity, finding that 33.8% of elderly patients had an affected relative, compared to 21.3% of adult patients [2]. In this study, the Hurley grading scale was used to compare the HS severity of the two groups, but no significant differences were observed [2]. In contrast, a cross-sectional study by Jiang et al. established a positive relationship between disease severity and age of onset. The main outcome was that the International Hidradenitis Suppurativa Severity Score System (IHS4) and the geometric mean ratio (GMR) were significantly higher in the group of late-onset patients than in the common-onset group [16]. The authors also compared other classifications, such as the Hurley stage and the Physician’s Global Assessment (PGA). Hurley stage III was present in 58% of patients with late-onset HS, compared to 28% of those with common-onset HS; in contrast, a ‘severe’ or ‘very severe’ PGA among the former was found in 37% of patients and among the latter in only 8% of cases [16]. BMI is reported to not particularly differ between elderly and adult HS patients, whereas it was significantly higher in elderly HS patients than in the age-matched controls [2]. Furthermore, elderly HS patients were more frequently ex-smokers than the adult HS group, with OR 2.035. In contrast, the latter were more commonly active smokers, with 38.4% of patients compared to 20.9% of the affected elderly [2]. Another aspect to be considered with regard to patients with HS tarda is that of comorbidities, which impact on the quality of life and treatment of these patients. According to van der Weijden et al., there was a significantly higher prevalence of psoriasis (20.1% vs. 7.9%), history of acne (16.7% vs. 4.8%), diabetes (9.2% vs. 4.8%), irritable bowel syndrome (71.4% vs. 56%), kidney disease (8.8% vs. 4.5%), chronic obstructive pulmonary disease (COPD) (14.1% vs. 8.1%) and fibromyalgia (45.1% vs. 27.2%) in the elderly patient group than in the adult HS group [2]. Another study of 1100 patients in the Italian population observed a higher prevalence of dermatitis (including atopic, seborrheic and perioral) among patients with late-onset HS than among early-onset HS (1.9% vs. 0.7%) [4]. This was also estimated for psoriasis (3.5% vs. 1.3%) and diabetes (1.2% vs. 0.2%) [4]. It must be underlined that the same study contradicts what is reported above; in fact, no significant differences were found in terms of disease location, with armpits, groin and buttocks comparably affected in both the early-onset and late-onset HS group [4]. In addition, a lower severity of disease was estimated in the late-onset group rather than its counterpart, as calculated with the Sartorius score [4]. The discrepancies are probably due to the different sample sizes and, above all, to the different division into groups compared with the work of van der Weijden et al. In fact, whereas in van der Weijden et al.’s study the cut-off was set at 60 years of age, in the work of Cazzaniga et al., the late teen years and age over 40 years are considered. In this regard, Nielsen et al. conducted a study of 700 patients with HS classifying them as van der Weijden et al. in over and under 60 years of age. Again, differences were found, such as a lower familiarity and higher frequency of buttock involvement in patients aged 60. Furthermore, the study estimated a higher severity of disease in the elderly patients using the IHS4 score, and they also found a positive correlation between age and IHS4 score in males [17].

### 3.3. Treatment

The treatment of HS in the elderly must necessarily take into consideration their frailty and the increased risk of multimorbidity that afflicts this category [2]. The European guidelines, however, do not report specific indications for this class of patients, but instead describe drugs contraindications. Their choice for elderly patients must therefore necessarily be based on their comorbidities. For example, TNFα inhibitors are contraindicated in patients with heart failure NYHA class III-IV, multiple sclerosis and severe infections. Polypharmacotherapy and possible drugs interactions must be considered in the elderly when selecting systemic antibiotics indicated for HS (tetracycline, clindamycin and/or rifampicin) [18]. For example, rifampin is a potent inducer of certain drug-metabolizing enzymes, such as cytochrome P450 (CYP) 3A4, and interacts with numerous drugs. In fact, it reduces the blood concentrations of beta-blockers and calcium antagonists, as well as various benzodiazepines [19]. For HS patients, however, secukinumab has received Food and Drug Administration (FDA) and European Medicines Agency (EMA) approval for HS. It is an anti-IL-17 not contraindicated in patients with heart failure; moreover, differently from anti-TNF, the risk of reactivation of latent tuberculosis seems to be a concern [20]. In addition, secukinumab has shown efficacy on HS in several studies. For instance, an Italian 52-Week Real-Life Study observed the achievement of Hidradenitis Suppurativa Clinical Response (HiSCR) score at 16 weeks in 57% of patients. Furthermore, from week 16 to 52, the HiSCR was achieved by 71% of the patients, and in all severity scales (IHS4, DLQI and VAS pain scale), the values showed a decrease [21]. However, according to guidelines and expert opinions, there is currently no reason not to consider systemic antibiotics as a first-line therapy in the elderly with HS [20]. The retrospective study by Denny et al. reinforces this concept by observing that in old age there are better responses to first-line therapies, such as topical and systemic antibiotics and intralesional corticosteroids [7]. On the other hand, a study by Prens et al. calculated drug survival, i.e., the measure of length of time until the discontinuation of a drug, in a population of adult patients with HS treated with biological agents; they found that older age, severity and duration of disease correlated positively with the survival of adalimumab and infliximab [22]. The authors explained this by hypothesizing that patients with a higher disease burden and who cared more about their health status, as older people do, had a greater compliance to accept long-term therapy such as anti-TNFα [22].

## 4. Discussion

Managing hidradenitis suppurativa (HS) in elderly patients presents a multifaceted challenge within dermatological practice, necessitating a comprehensive understanding of the disease’s nuances, treatment modalities and the unique physiological and clinical characteristics of older individuals. With HS being a chronic inflammatory condition primarily affecting the anogenital and intertriginous regions, its management in the elderly requires a tailored approach that considers not only the dermatological manifestations but also the impact of age-related changes on treatment efficacy and tolerability [23,24,25,26,27,28,29,30,31,32,33,34,35].

One of the key considerations in managing HS in older patients is the recognition of the disease’s atypical presentation and its potential overlap with other dermatological conditions commonly seen in this population. Clinical features such as the involvement of different anatomical sites, including the neck, mammary region and lower extremities, as well as variations in lesion morphology and distribution, may pose diagnostic challenges and necessitate a high index of suspicion for HS in elderly individuals [27,28,29,30].

Furthermore, the epidemiological profile of HS in the eldRingerly differs from that of younger cohorts, with studies suggesting a lower prevalence of the disease but a higher average age of onset. The categorization of HS tarda, defined as disease onset after the age of 60, underscores the importance of recognizing age-related variations in disease presentation and progression. Additionally, the shift in the female-to-male ratio observed in older patients may reflect the influence of hormonal changes, such as menopause, on disease pathogenesis and clinical course.

Understanding the interplay between age-related comorbidities and HS is paramount to optimizing treatment outcomes in elderly patients. Conditions such as obesity, diabetes mellitus and COPD are prevalent in this population and may impact both the severity of HS and the choice of therapeutic interventions. These comorbidities very often are associated with HS, such as obesity, but at the same time are independent conditions that affect elderly patients.

Moreover, the potential for drug–drug interactions and the need to consider the overall burden of polypharmacy highlight the importance of a holistic approach to treatment decision-making in elderly patients with HS [6].

In terms of therapeutic interventions, while systemic antibiotics remain a cornerstone of treatment for HS, particularly in the setting of acute flares and mild-to-moderate disease, the use of biologic agents such as TNFα inhibitors and secukinumab has emerged as a promising option for refractory cases. However, the safety and efficacy of these agents in elderly patients, particularly those with underlying cardiac comorbidities, warrant careful consideration and may necessitate individualized treatment strategies [31,32,33,34,35,36,37,38,39,40,41,42,43,44,45,46,47,48].

Although the current literature on HS does not allow us to establish with certainty the efficacy and safety of biologics, it is important to point out that, in contrast, for conditions such as psoriasis, the efficacy and safety of these drugs have been well-documented and demonstrated for many years. This extensive positive experience with biologics in the treatment of psoriasis provides us with a valuable foundation and allows us to approach their use in HS with a degree of optimism. Indeed, the successful approval and widespread adoption of biologics for psoriasis serve as a significant reference point [40,41,42]. This suggests that the benefits observed in psoriasis patients could potentially be mirrored in HS patients as well, pending further research and clinical trials. The hope is that, as research progresses and more clinical trials are conducted, similarly positive outcomes will be achieved for HS. This would significantly enhance the therapeutic options and improve the quality of life for those suffering from this condition.

A comprehensive approach to managing HS in elderly patients should encompass not only pharmacological interventions but also lifestyle modifications, wound care and surgical options where appropriate. Moreover, given the chronic and relapsing nature of HS, ongoing monitoring and regular follow-up are essential to assess treatment response, manage disease flares and address any emerging concerns or complications.

## 5. Conclusions

In conclusion, managing HS in elderly patients requires a nuanced understanding of age-related changes in disease presentation, comorbidity profiles and treatment responses. A multidisciplinary approach involving dermatologists, geriatricians and other specialists is crucial for tailoring treatment strategies to the unique needs of this patient population and optimizing long-term outcomes and quality of life.

## Data Availability

Data are reported in the current study.
